# Effectiveness of neutrophil-to-lymphocyte and platelet-to-lymphocyte ratios in predicting the incidence of nausea and vomiting after total knee arthroplasty in patients with hemophilia A

**DOI:** 10.3389/fsurg.2023.1120930

**Published:** 2023-04-17

**Authors:** Denghe Feng, Dong Wang, Changping Gu, Meng Lv, Zaibo Liu, Yuelan Wang

**Affiliations:** ^1^ School of Anesthesiology, Weifang Medical University, Weifang, China; ^2^ Department of Anesthesiology, The First Affiliated Hospital of Shandong First Medical University & Shandong Provincial Qianfoshan Hospital, Shandong Institute of Anesthesia and Respiratory Critical Medicine, Jinan, China

**Keywords:** neutrophils, lymphocytes, platelets, hemophilia, postoperative nausea and vomiting

## Abstract

**Objective:**

To investigate the ability of preoperative neutrophil-to-lymphocyte ratio (NLR) and platelet-to-lymphocyte ratio (PLR) to predict postoperative nausea and vomiting (PONV) after total knee arthroplasty (TKA).

**Methods:**

The clinical data of 108 male patients with hemophilia A who underwent TKA an our institution were collected and analyzed. Confounding factors were adjusted by propensity score matching. The best cutoffs of the NLR and PLR were determined by the area under the receiver operating characteristic curve (ROC). The predictive ability of these indexes was assessed by measuring the sensitivity, specificity, and positive and negative likelihood ratios.

**Results:**

There were significant differences in the use of antiemetics (*p* = 0.036) and the incidence of nausea (*p* < 0.001) and vomiting (*p* = 0.006) between the two groups (NLR <2 and ≥2). An increase in preoperative NLR was an independent risk factor for PONV in patients with hemophilia A (*p* < 0.05). ROC analysis showed that NLR significantly predicted the occurrence of PONV (cutoff value: 2.20, ROC: 0.711, *p* < 0.001). In turn, the PLR did not strongly predict PONV.

**Conclusions:**

The NLR is an independent risk factor for PONV in patients with hemophilia A and can significantly predict this event. Thus, follow-up monitoring is essential for these patients.

## 1. Introduction

Hemophilic arthropathy is a debilitating complication of hemophilia, leading to intra-articular hemorrhage, especially in knee joints (1). Total knee arthroplasty (TKA) is the standard treatment for end-stage hemophilic arthropathy (2). General anesthesia is the preferred anesthetic method for patients with hemophilia because the risk of bleeding is lower than that of neuraxial anesthesia. However, general anesthesia causes postoperative nausea and vomiting (PONV) in 20%–30% of surgical patients (3). The neutrophil-to-lymphocyte ratio (NLR) and platelet-to-lymphocyte ratio (PLR) are used for the diagnosis and follow-up of inflammatory diseases (4). The mechanism that causes nausea has not been studied to a large extent. The vomiting reflex seems to originate from the vomiting center located in the dorsolateral medulla oblongata. Inflammation is known to increase the risk of PONV (5). However, the ability of these indexes to predict PONV in patients with hemophilia has not been assessed. This study retrospectively analyzed the clinical data of patients with hemophilia A and assessed the ability of preoperative NLR and PLR to predict PONV after TKA.

## 2. Methods

### 2.1. Study design

This retrospective study included 108 male patients who underwent TKA at the First Affiliated Hospital of Shandong First Medical University, China, from March 2018 to December 2021. The study was approved by the Research Ethics Committee of our institution [XMSB-LL-2022(044)]. The trial was registered at chictr.org.cn on January 27, 2021 (Registration No: NCT05636163; principal investigator: YW).

### 2.2. Inclusion and exclusion criteria

The inclusion criteria were (1) patients with hemophilia A who underwent unilateral total knee replacement, (2) age of 18–70 years, (3) American Society of Anesthesiologists (ASA) physical status I–III, (4) no history of mental illness, (5) no history of use of antiemetics and anticholinergic drugs, and (6) no history of serious gastrointestinal diseases. The exclusion criteria were (1) patients who received long-term steroid therapy before surgery, (2) patients requiring more than 2.5 mg of neostigmine to reverse neuromuscular blockade, and (3) patients with prior malignancy, malabsorption, morbid obesity, hypogonadism, thyroid and parathyroid diseases, and autoimmune diseases. All patients ceased the intake of solid foods and fluids 6 and 2 h before surgery, respectively. All surgeries were performed by the same group of surgeons.

### 2.3. Primary and secondary outcomes

The primary outcome was the incidence of PONV, which was defined as the occurrence of nausea or vomiting within 24 h after surgery (5). The secondary outcomes were (1) the dosage of postoperative antiemetics (ondansetron, metoclopramide, and dexamethasone), (2) postoperative visual analog scale scores immediately after returning to the ward, and (3) the use of patient-controlled analgesia (PCA).

### 2.4. Sample size calculation

The incidence of PONV was expected to be 30.4% and 67.4% in the group with NLR <2 and ≥2, respectively, with *α* of 0.05 and power of 90%. The required sample size was calculated using PASS version 15.0 (NCSS, LLC. Kaysville, Utah, USA). The study required a minimum of 34 patients in each group, and the sample size was increased to 43 patients per group to compensate for the loss to follow-up.

### 2.5. Patient allocation and data collection

A total of 108 male patients with hemophilia A who underwent TKA under general anesthesia at our institution were selected and divided into two groups according to NLR values (<2 and ≥2), as described previously (6, 7). Data on age, ASA physical status, smoking history, alcohol consumption, and underlying diseases were collected.

The following data were collected from electronic medical records: sex, age, ASA physical status, body mass index (BMI), past medical history, length of surgery, preoperative neutrophil and lymphocyte count, NLR, PLR, and the use of antiemetic medications (ondansetron, metoclopramide, and dexamethasone).

In detail, the inflammatory markers were analyzed, calculating the NLR and PLR. The NLR and PLR were obtained by dividing the absolute neutrophil and platelet counts by the lymphocyte count.

### 2.6. Statistical analysis

Statistical analyses were performed using SPSS version 25.0 (IBM, Chicago, IL, USA) and MedCalc version 20.1.0 (MedCalc Software, Ostend, Belgium). The following factors were adjusted by propensity score matching: age, ASA classification, BMI, smoking history, alcohol consumption, hypertension, diabetes mellitus, and allergy medications (6).

Normally distributed continuous variables were expressed as mean ± standard deviation and compared using a *t*-test. Non-normally distributed continuous variables were expressed as median (quartiles) [M (Q1, Q3)] and compared using the Mann–Whitney *U* test. Categorical variables were expressed as percentages and compared using the chi-squared test or Fisher's exact test. Independent risk factors in the univariate analysis (*p *< 0.05) were expressed as odds ratios and corresponding 95% confidence intervals (CIs). The predictive value of NLR and PLR for PONV was assessed by the area under the receiver operating characteristic curve (ROC). Two-sided *p-*values of <0.05 were considered statistically significant.

## 3. Results

### 3.1. Baseline characteristics

There were no significant differences in baseline data between the two groups after adjusting for confounders. Additionally, there were no significant intergroup differences in the use of PCA, tourniquets, or ultrasound-guided saphenous nerve block (*p *> 0.05) (Table 1).

**Table 1:** Comparison of baseline characteristics and surgical information between the two groups of patients.

		Group1 (NLR < 2)	Group 2 (NLR ≥ 2)	*p*-value
*N*		43	43	
Age	Years	31.28 ± 8.81	31.77 ± 7.93	0.825a
BMI		22.81 ± 4.14	22.83 ± 3.96	0.983a
Operation time	Hours	2.15 ± 0.76	2.41 ± 0.85	0.149a
VAS		2.63 ± 1.69	2.40 ± 1.35	0.397b
ASA	II	29	30	1.000c
	III	14	13	
Smoking	Yes	0	1	1.000c
	No	43	42	
Alcohol	Yes	0	1	1.000c
	No	43	42	
Hypertension	Yes	3	3	1.000c
	No	40	40	
Diabetes	Yes	0	0	1.000c
	No	43	43	
Allergy	Yes	9	10	1.000c
	No	34	33	
PCA	Yes	17	20	0.663c
	No	26	23	
Tourniquet	Yes	31	29	0.815c
	No	12	14	
Saphenous nerve block	Yes	7	7	1.000c
	No	36	36	

BMI, body mass index; PCA, patient controlled analgesia; a, paired two-sample t-test; b, Wilcoxon signed rank sum test; c, chi-square test.

### 3.2. Postoperative use of antiemetics and incidence of PONV

There were significant differences in the postoperative use of antiemetics and the incidence of nausea and vomiting between the two groups (NLR ≥ 2 and NLR < 2) (*p *= 0.036, *p *< 0.001, and *p *= 0.006, respectively) (Table 2). Five (11.6%) and fourteen (32.6%) patients in the group with NLR < 2 and NLR ≥ 2, respectively, used antiemetic medications. In the group with NLR < 2, 6 (14.0%) patients had nausea or vomiting, and 37 (86.0%) had none of these complications. In the group with NLR ≥ 2, 24 (55.8%) patients had nausea or vomiting, and 19 (44.2%) had none of these complications (Table 2).

**Table 2:** Comparison of postoperative use of antiemetics and incidence of PONV in two groups.

		Group 1 (NLR < 2)	Group 2 (NLR ≥ 2)	*p*-value
*N*		43	43	
Antiemetic use in 24 h	Yes	5	14	0.036
	No	38	29	
Nausea	Yes	6	24	<0.001
	No	37	19	
Vomiting	Yes	5	17	0.006
	No	38	26	

### 3.3. Analysis of factors influencing the occurrence of PONV

The poor prognosis of patients with PONV was considered the dependent variable, and age, ASA physical status, length of surgery, BMI, use of PCA and tourniquets, ultrasound-guided saphenous nerve block, preoperative NLR, and PLR level from lowest to highest were assigned in the univariate logistic regression analysis. The results showed that increased preoperative NLR was an independent risk factor for the development of PONV (*p *< 0.05) (Table 3).

**Table 3:** Univariate logistic regression analysis of factors associated with the occurrence of PONV in patients with hemophilia A.

		Odds ratio	95% confidence interval	*p*-value
Age	Years	0.992	0.927–1.063	0.828
BMI		0.950	0.828–1.090	0.465
Operation time	Hours	1.421	0.685–2.946	0.345
NLR		2.603	1.047–6.472	0.040
PLR		0.999	0.985–1.013	0.861
PCA		1.660	0.499–5.516	0.408
Tourniquet		1.988	0.501–7.889	0.329
Saphenous nerve block		1.758	0.412–7.490	0.446

NLR, neutrophil-to-lymphocyte ratio; PLR, platelet-to-lymphocyte ratio.

### 3.4. ROC analysis of NLR and PLR

**Figure 1:**
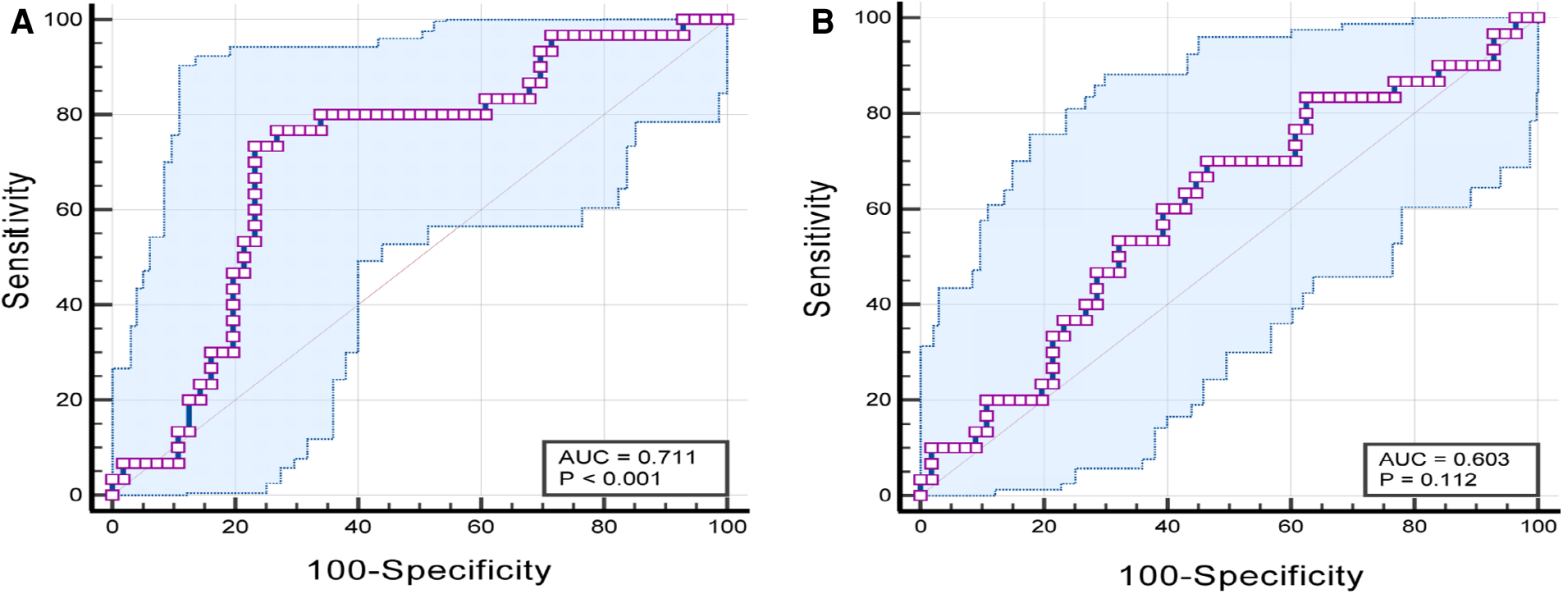
(**A**) ROC curve of PONV diagnosis predicted by NLR parameters; (**B**) ROC curve of PONV diagnosis predicted by PLR parameters. ROC, receiver operating characteristic curve; PONV, postoperative nausea and vomiting; NLR, neutrophil-to-lymphocyte ratio; PLR, platelet-to-lymphocyte ration..

The ROC showed that NLR could significantly predict PONV (ROC: 0.711, 95% CI: 0.603–0.803, *p *< 0.001). The Youden index was highest (0.5012) at an NLR > 2.20, with a sensitivity of 0.733 (95% CI: 0.541–0.877), specificity of 0.770 (95% CI: 0.636–0.870), positive likelihood ratio of 3.16, and negative likelihood ratio of 0.35. PLR could not significantly predict PONV (ROC: 0.603, 95% CI: 0.492–0.707, *p* = 0.112) (Figures 1A,B).

## 4. Discussion

In this study, laboratory indicators NLR and PLR were collected to determine whether they could predict the occurrence of nausea and vomiting in patients with hemophilia. The study found that NLR was an independent risk factor for PONV after adjusting for confounders. The ROC analysis showed that NLR could accurately predict the occurrence of PONV.

The centers of the autonomic nervous system associated with vomiting are at the level of the medulla oblongata in the hindbrain. Chemo-sensitive receptors detect substances in the blood and transmit this information to the adjacent nucleus of the solitary tract in the hindbrain region (8). Inflammatory mediators may affect nausea and vomiting centers through blood circulation. In previous literature, studies have used NLR and PLR as inflammatory parameters to determine the prognosis of some diseases, such as heart disease, vascular disease and tumor (9–11). The NLR is a parameter for the diagnosis and follow-up of inflammatory diseases and surgical outcomes. Additionally, NLR is a marker for PONV in patients undergoing maxillofacial surgery (7). Tayfur et al. evaluated the relationship between the platelet-lymphocyte ratio and hyperemesis gravidarum in 433 patients and 160 controls (12). NLR and PLR can predict PONV in patients undergoing breast reduction surgery (13). A PLR cutoff of 137.2 could significantly predict PONV, and the sensitivity of this PLR value for predicting PONV was higher than that of NLR (77.8% vs. 73.3%). The ROC analysis showed that the ability of PLR to predict PONV was higher than that of NLR. In our cohort, preoperative NLR with a cutoff of 2.20 significantly predicted the occurrence of PONV; however, preoperative PLR did not accurately predict PONV in our patients. This result may be related to the small sample size. Thus, large prospective studies are needed to confirm this relationship.

PONV is a common postoperative symptom. The incidence of PONV is 30%–50% in surgical patients and up to 80% in high-risk patients (14, 15). Hemophilia is characterized by reduced levels of coagulation factors (16). Gastrointestinal bleeding is probably the most challenging event in hemophiliacs. Vascular dysplasia along with postoperative vomiting is likely to trigger gastrointestinal bleeding when coagulation factors are deficient or have reduced activity (17). Therefore, this study assessed PONV in these patients.

Ondansetron is the most commonly used 5-hydroxytryptamine receptor antagonist and is the gold standard for PONV therapy (Evidence A1) (18). The United States Food and Drug Administration recommends doses smaller than 16 mg for chemotherapy-induced nausea and vomiting because of the risk of prolongation of the QT interval (19). Metoclopramide has antiemetic effects at doses larger than 20 mg (20). In our study, based on follow-up and historical recommendation two of the patients who experienced vomiting were given 10 mg of metoclopramide with poor results, and the addition of 8 mg of ondansetron relieved symptoms, supporting the evidence for increased metoclopramide use.

Achieving adequate analgesia after TKA is challenging, and different multimodal analgesic regimens are effective (21). We can use PCA or ultrasound-guided saphenous nerve blocks for postoperative analgesia, but saphenous nerve blocks carry the risk of puncturing blood vessels and are not conducive to postoperative recovery. In our cohort, PCA was used by 37 patients postoperatively. Postoperative opioid use is a strong predictor of PONV development (3). Various antiemetic drugs reduce PONV (22), so we routinely use ondansetron 8 mg for this purpose. In our cohort, 30 patients had vomiting, and the incidence of PONV was significantly higher in the group with NLR > 2. The current evidence for recommending two or more antiemetics for preventing PONV is strong, and combination therapy is superior to monotherapy (23).

This study has limitations. First, we analyzed data from a single center, the sample size was small, and the study was retrospective in nature. Second, preoperative nausea and vomiting symptoms were based on patient-reported responses, and the incidence of symptoms might be underestimated because of recall bias. However, adjusting confounding factors for postoperative outcomes by propensity score matching reduced the bias in this study.

## 5. Conclusion

NLR has good ability to predict PONV after TKA in patients with hemophilia A. Thus, blood parameters should be analyzed to improve surgical outcomes in these patients. However, this result needs to be confirmed by larger studies.

## Data Availability

The original contributions presented in the study are included in the article/Supplementary Material, further inquiries can be directed to the corresponding author.
